# Bibliometric and visualized analysis on global trends and hotspots of TAK1 in regulated cell death: 1999 to 2024

**DOI:** 10.3389/fimmu.2024.1437570

**Published:** 2024-10-15

**Authors:** Kun Huang, Ye He, Hao Wan, Xiao-Xia Ban, Xin-Yu Chen, Xi-Min Hu, Xin-Xing Wan, Rui Lu, Qi Zhang, Kun Xiong

**Affiliations:** ^1^ Department of Human Anatomy and Neurobiology, School of Basic Medical Science, Central South University, Changsha, China; ^2^ Xiangya School of Medicine, Central South University, Changsha, China; ^3^ Changsha Aier Eye Hospital, Changsha, China; ^4^ Department of Endocrinology, Third Xiangya Hospital, Central South University, Changsha, China; ^5^ Department of Molecular and Cellular Physiology, Stanford University, Stanford, CA, United States; ^6^ Department of Ophthalmology, Stanford University School of Medicine, Palo Alto, CA, United States; ^7^ Key Laboratory of Emergency and Trauma of Ministry of Education, College of Emergency and Trauma, Hainan Medical University, Haikou, China; ^8^ Hunan Key Laboratory of Ophthalmology, Changsha, China

**Keywords:** TAK1, bibliometric analysis, CiteSpace, VOSviewer, regulated cell death, necroptosis, PANoptosis, cancer

## Abstract

**Background:**

Regulated cell death (RCD) is a genetically controlled form of cell death that plays an important role in organogenesis, tissue remodeling, and pathogenesis of cancers. Transforming growth factor-beta-activation kinase 1 (TAK1) is a member of the serine/threonine protein kinase family, which can respond to internal and external stimuli and participate in inflammatory responses through multiple signaling pathways and cellular processes. In the last two decades, the regulatory roles of TAK1 at the crossroads of multiple RCD pathways, including apoptosis, necroptosis, pyroptosis, and PANoptosis were revealed by 801 articles retrieved from the Web of Science Core Collection database. To analyze global research trends and hotspots concerning the role of TAK1 in RCD, the bibliometric and visualized analysis were applied in the current study.

**Methods:**

The data for this bibliometrics study were retrieved from the Web of Science Core Collection database. The search formula was (TS=(Apoptosis) OR TS=(pyroptosis) OR TS=(Necroptosis) OR TS=(PANoptosis) OR TS=(Autophagy) OR TS=(Ferroptosis) OR TS=(cuproptosis)) AND ((TS=(TAK1)) OR TS=(MAP3K7)). The co-occurrence and co-cited analysis on basic bibliometric parameters were conducted by VOSviewer. The dual-map overlay of journals, citation bursts, keyword timelines, and keyword bursts were analyzed by CiteSpace.

**Results:**

A total of 801 articles from 46 countries have been included in the analysis. The number of publications demonstrates a consistent increase from 1999 to 2024. The primary research institutions driving this field are Osaka University Notably, the Journal of Biological Chemistry stands out as the most popular journal in this domain. These publications collectively involve contributions from 4663 authors, with Jun Tsuji emerging as a prolific author. Jun Tsuji also gains the highest co-citation frequency. Emerging research hotspots are encapsulated by keywords, including apoptosis, NF-κB, inflammation, autophagy, and TNFα.

**Conclusion:**

This is the first bibliometric and visualized study to analyze the global trends and hotspots of TAK1 in RCD. Based on the analysis of 801 articles, the results provide a retrospective and comprehensive visualized view of the research hotspots and frontiers of TAK1 at the crossroads of multiple RCD signaling pathways and propose ideas for guiding their future investigations in molecular mechanisms and therapeutic strategies in this field.

## 1 Introduction

Transforming growth factor-β (TGF-β) activated kinase 1 (TAK1) is a serine/threonine kinase belonging to the mitogen-activated protein tri-kinase (MAP3K) family, also known as MAP3K7. TAK1 was first identified as a TGF-β-activated kinase (1), and over the years it has been shown to respond to a number of internal and external stimuli to phosphorylate a wide range of downstream targets and trigger diverse cellular responses in a variety of diseases, including cancers and neurodegenerative diseases (2–5). TAK1 forms a complex with TAK1-binding proteins (TAB1 and TAB2/TAB3) that is essential for TAK1 activation (6–8). Currently, TAK1 is considered a key component of the NF-κB and mitogen-activated protein kinase (MAPK) signaling pathways (9, 10).

Regulated cell death (RCD) widely exists in the development of organisms, and is a genetically regulated active, and orderly way of cell death (11–14). RCD includes apoptosis, pyroptosis, autophagy, necroptosis, PANoptosis, and other pathways, which are necessary for the renewal of cells and the maintenance of their homeostasis, and are also effective self-protection pathways when the organism is subjected to external stresses (15–18). RCD is beneficial under certain physiological circumstances, for example, it can maintain the normal function of the organism by mediating apoptosis, a non-inflammatory RCD, of infected pathogen cells (14, 19–21). Also, RCD is involved in various human diseases (22). Spontaneous cell death at different stages of growth and development can maintain stable genetic traits (23, 24). However, immune dysregulation occurs with occurrences of some inflammatory RCDs, such as pyroptosis and necroptosis, which can be manifested as severe autoimmunity in the body (25). RCD dysregulation can also lead to neurological abnormalities, such as pyroptosis has been found to promote the progression of multiple sclerosis (MS) (26). PANoptosis, a newly defined integral form of RCD, combines features of three major RCD pathways: pyroptosis, apoptosis, and necroptosis (20). The identification of PANoptosis provides a potential to elucidate the complex scenarios of cell death in diverse pathological conditions (21). RCD can be triggered in response to a variety of events including inflammation, tissue injury, and developmental processes (27–31). Deletion of TAK1 activates multiple pathways *via* TNFα to regulate RCD. Increasing evidence also suggests that TAK1 is essential for cell survival (32, 33).

Bibliometrics is a newly developed powerful tool that applies mathematical and statistical methods to assess the hotpots and trends of a tremendous amount of literature data in the scientific field (34). It can help researchers not only quickly comprehend research hotspots and trends in a certain research topic, but also examine the distribution of countries/regions, authors, and journals in the research area, establishing the groundwork for future research orientations and development (35). For example, Francesca et al. utilized bibliometric analysis to present a thorough examination of the scientific literature on immunonutrition and cancer research, offering valuable insights into enhancing cancer care practices through evidence-based nutritional interventions (36). Using bibliometric analysis, Wang et al. highlights the cellular and molecular pathway mechanisms of pyroptosis, shedding light on its pivotal role in oncogenesis, progression, and treatment (37). There are more than eight hundred papers related to TAK1 have been published, and it is hard to identify emerging hotspots and research trends in this field through a traditional literature review. Thus, the purpose of this study is to investigate research hotspots and development patterns in the field of TAK1 in RCD, as well as to map scientific information with CiteSpace and VOSviewer to propose new ideas for its research status and future trends.

## 2 Materials and methods

### 2.1 Data retrieval and collection

**Figure 1:**
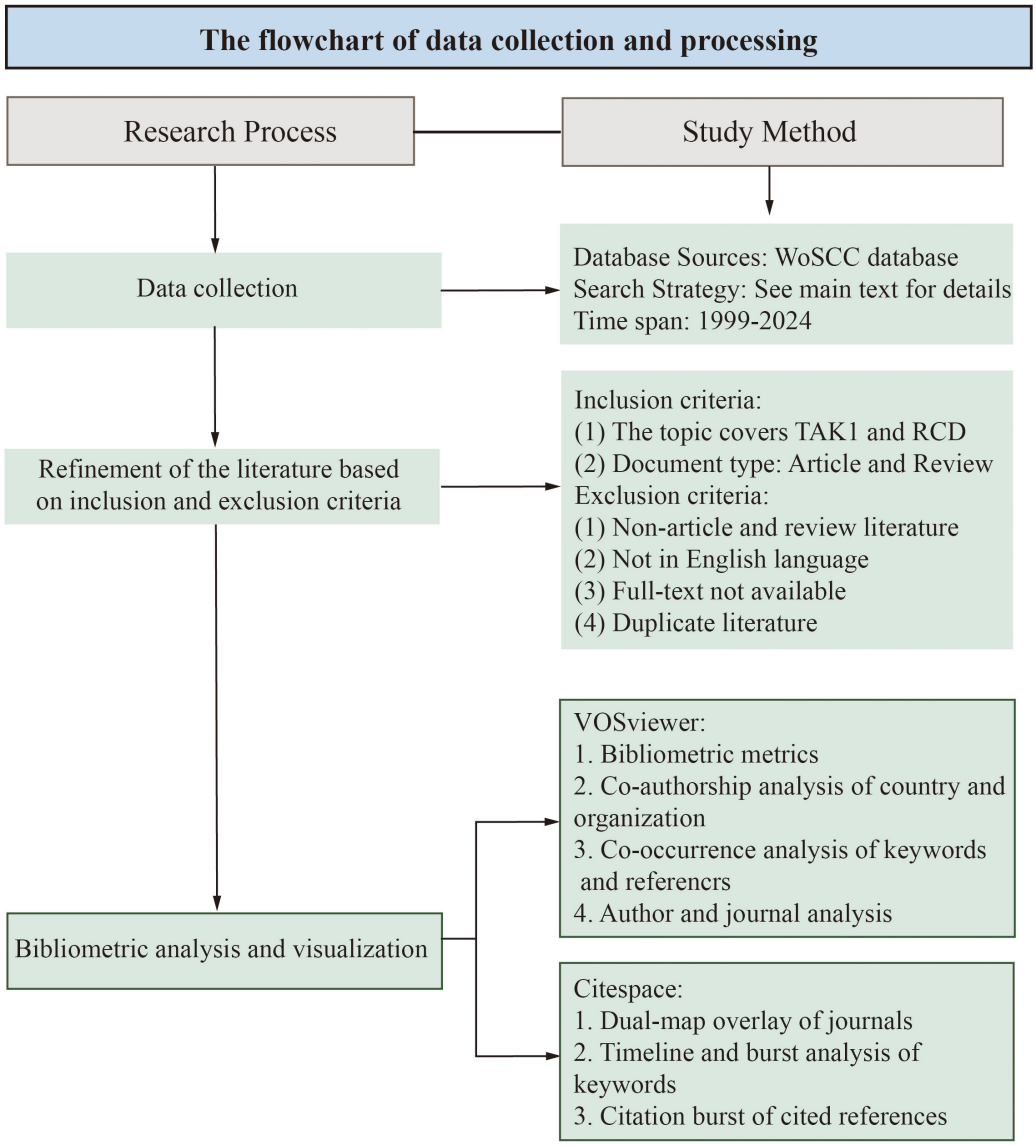
The flowchart of data collection and processing.

The data for this bibliometrics study were collected from the Web of Science Core Collection (WoSCC) database, with Science Citation Index Extended (SCIE), Social Science Citation Index (SSCI) and Emerging Sources Citation Index (ESCI) included. The data were retrieved from the WoSCC database on April 15, 2024. The search formula was (TS=(Apoptosis) OR TS=(pyroptosis) OR TS=(Necroptosis) OR TS=(PANoptosis) OR TS=(Autophagy) OR TS=(Ferroptosis) OR TS=(cuproptosis)) AND ((TS=(TAK1)) OR TS=(MAP3K7)). A total of 845 documents were retrieved from the WoSCC database. After further selection of the types and language of articles used for the bibliometric analysis, we obtained a total of 801 papers for the bibliometric analysis. The language was limited to “English” and the types were limited to “Article” and “Review” (**Figure 1**).

### 2.2 Bibliometric analysis

VOSviewer is a bibliometric tool that enables the mapping of scientific knowledge through the construction and visualization of relationships in network data, showing the structure, evolution, and cooperation of knowledge fields for large-scale data (38). CiteSpace supports a wide range of processes to help visualize and analyze the domain’s structure, dynamic patterns, and trends. The size of the nodes in the CiteSpace visualization denotes the frequency of co-occurrence, while the connecting lines show interrelationships (39–41). Bibliometric analysis and visualization were conducted with VOSviewer (version 1.6.18; https://www.vosviewer.com/download) and Citespace (version 6.1.R6; https://sourceforge.net/projects/citespace/files). In our study, we used VOSviewer to complete country and organization analysis, journal analysis, author and co-cited author analysis, reference co-occurrence analysis, and keyword co-occurrence analysis. Meanwhile, CiteSpace was used to analyze the dual-map overlay of journals, citation bursts, keyword timelines, and keyword bursts. Additionally, we used Graphpad Prism 8.0 (https://www.graphpad.com/) to analyze the annual publications.

## 3 Results

### 3.1 The trend of annual publications

**Figure 2:**
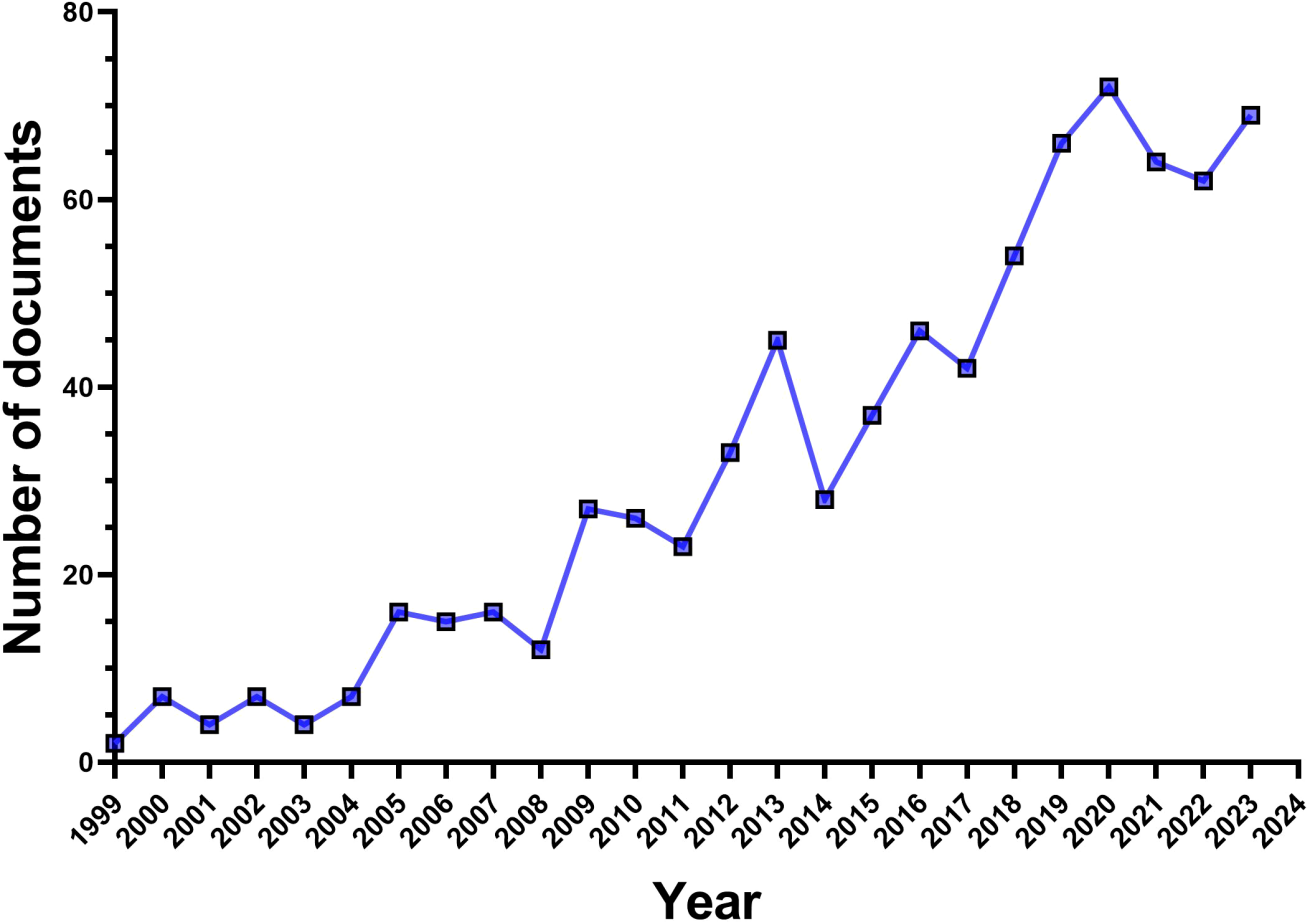
Number of publications on TAK1 in the field of RCD by year.

According to our retrieval strategy, there are a total of 801 publications of TAK1 in the field of RCD over the last two decades. The number of papers published between 1999 and 2024 exhibited a rapidly increasing trend (**Figure 2**). The annual number of publications before 2005 was less than 10, indicating that the field had begun to gain attention but had not yet been extensively studied. After 2005, the number of relevant publications per year began to increase rapidly. In 2020, 72 articles have been published.

### 3.2 Distributions of countries/regions and organizations

A total of 801 documents were published from 46 different countries/regions. The top 10 most prolific countries/regions with publications on TAK1 in the field of RCD are listed in **Table 1**. The country with the highest number of publications is China (n=366, 45.69%), followed by the United States (n=260, 32.46%), and Japan (n=86, 10.74%).

**Table 1:** Top 10 most productive countries/regions.

Rank	Country	Documents	Citations	Total link strength
1	China	366	9128	1481
2	USA	260	19390	2356
3	Japan	86	5076	1226
4	Germany	49	3501	524
5	South Korea	35	961	167
6	England	33	1242	399
7	India	19	712	149
8	Italy	17	2204	229
9	France	16	1004	199
10	Canada	15	1520	127

The combined number of publications from the three countries accounted for almost 90 percent of the total. The US has much more citations than other countries/regions.

**Figure 3:**
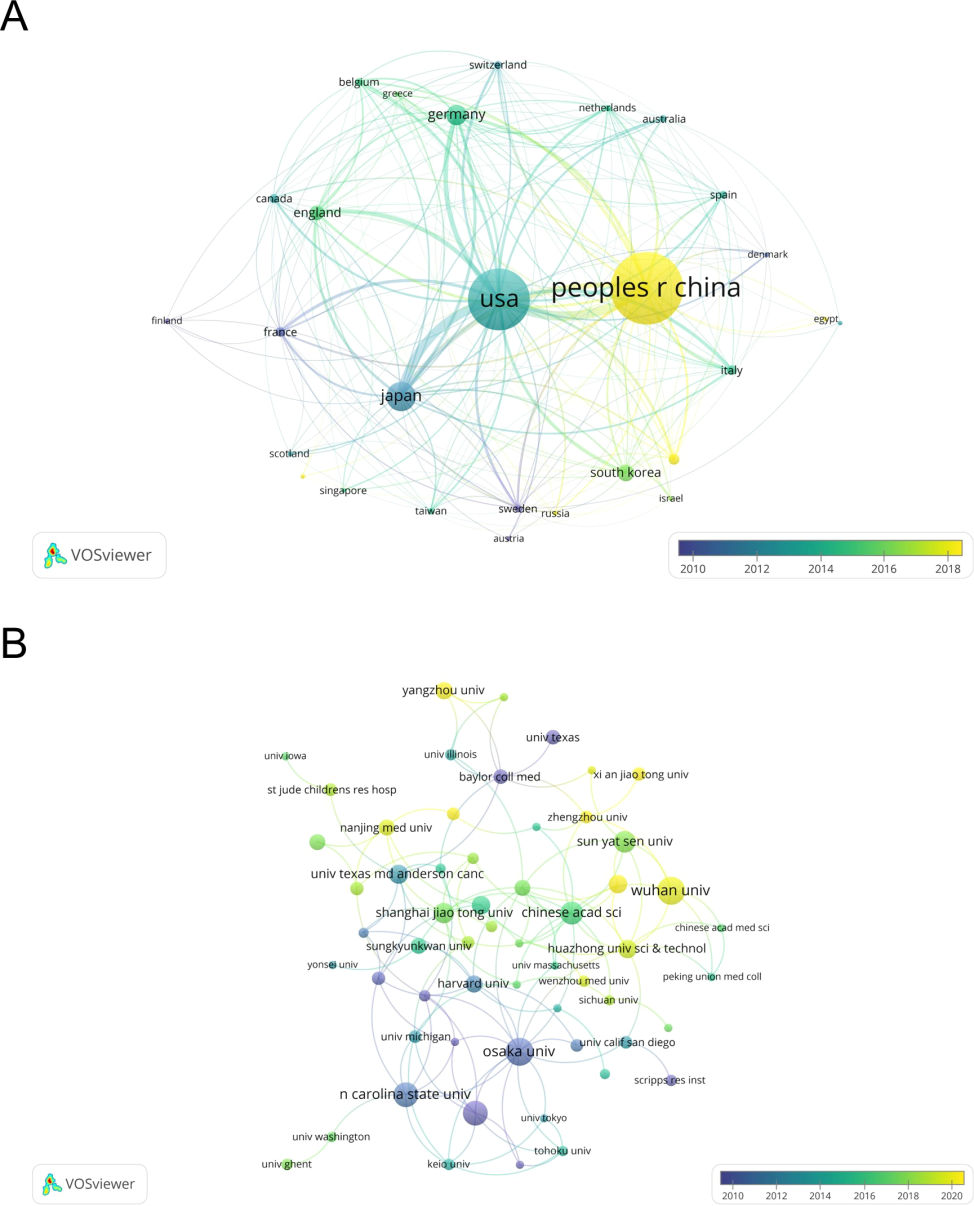
Distribution of publications from different countries/regions and organizations. **(A)** Distribution of publications from different countries/regions. **(B)** Distribution of publications from different organizations. The thickness of the connecting lines between the nodes reflects the strength of the interaction. The color of the node represents the average year of publication for the country which the node represents.

Then, we built a collaborative network based on the number of publications and relationships in each country/region, which visualized 24 countries/regions with greater than or equal to 4 publications (**Figure 3A**). The thickness of the connecting lines between the nodes reflects the strength of the interaction. There is close cooperation between the countries, with the United States at the core of the network of relationships. The United States and China, as well as the United States and Japan, have the closest cooperation. The color of the node represents the average year of publication for the country which the node represents. It can be seen that the node for China is in yellow, which indicates that the center of gravity of TAK1 research in RCD is gradually tilting toward China.

The 801 documents being analyzed originate from 1026 organizations. **Table 2** displays the 15 most productive institutions, along with the countries in which they are situated, the total number of citations, and the overall strength of their connections with each other. The most productive institutions are Osaka University and Wuhan University. Osaka University (2282), Nagoya University (2010), and Harvard University (1212) are the institutions with the most citations. **Figure 3B** illustrates the network of collaboration between the institutions. The nodes in yellow imply that these institutions are emerging stars in this bibliometric analysis; the purple color implies that these institutions have less research in this field. As can be observed, the Chinese organizations among the Top 15 most productive have continued to investigate this field in recent years.

**Table 2:** Top 15 most productive organizations.

Rank	Organization	Country	Documents	Citations	Total link strength
1	Wuhan University	China	27	536	42
2	Osaka University	Japan	22	2282	266
3	Nagoya University	Japan	19	2010	233
4	North Carolina State University	USA	19	1010	250
5	Chinese Academy of Sciences	China	18	1078	39
6	Sun Yat-sen University	China	17	899	36
7	Huazhong University of Science and Technology	China	15	538	60
8	Shanghai Jiao Tong University	China	15	311	32
9	The University of Texas MD Anderson Cancer Center	USA	14	936	82
10	Zhejiang University	China	14	352	22
11	Nanjing University	China	13	413	58
12	Nanjing Medical University	China	13	205	42
13	Yangzhou University	China	13	181	19
12	Harvard University	USA	12	1212	45
15	Nantong University	China	12	162	38

### 3.3 Authors and co-cited authors

**Figure 4:**
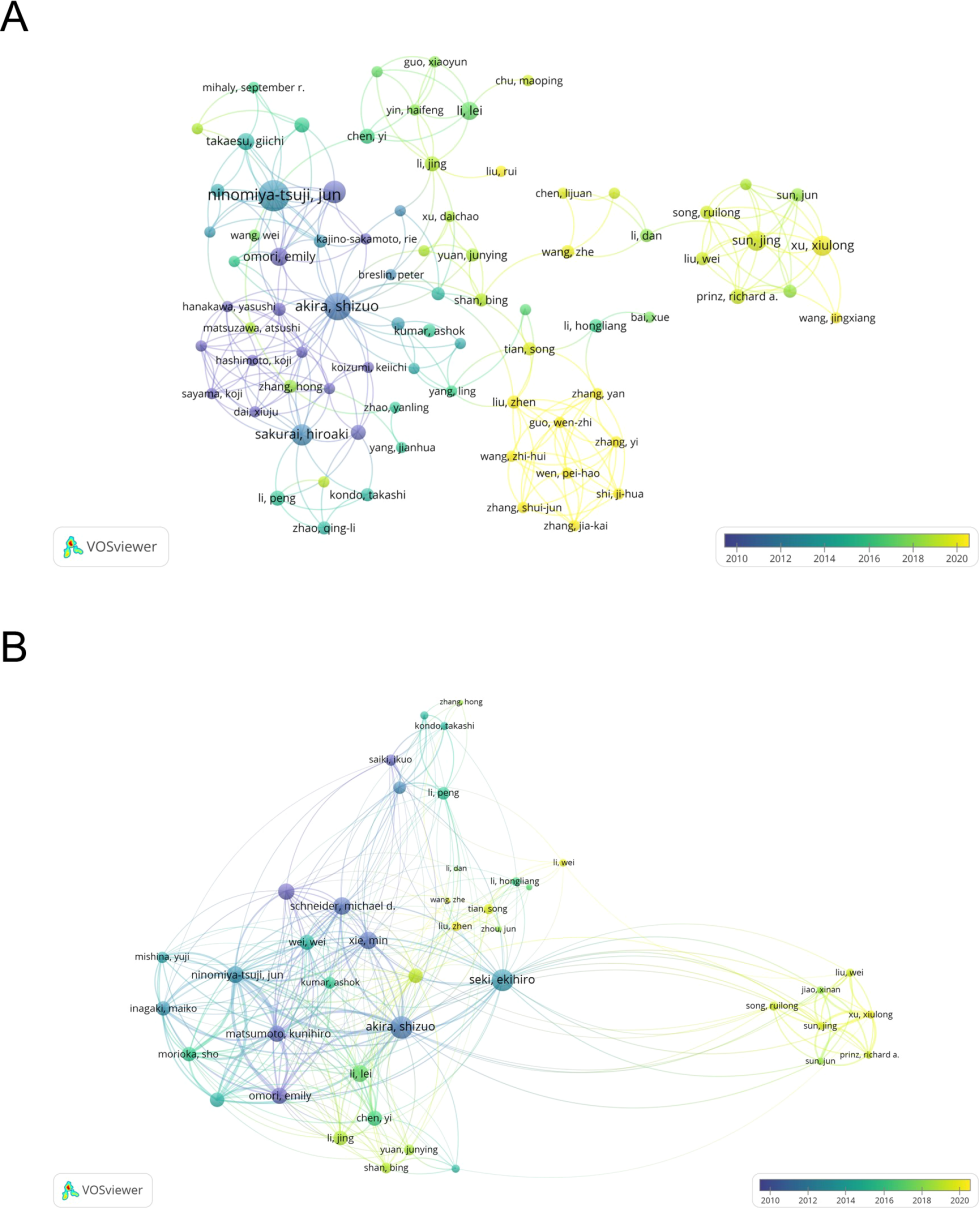
The map of co-authors and collaborative network. **(A)** The co-occurrence author’ map of co-authors in VOSviewer. **(B)** Analysis of collaborative network visualization of authors’ citations. The thickness of the connecting lines between the nodes reflects the strength of the interaction. The color of the node represents the average year of publication for the country which the node represents.

A total of 4663 authors have been involved in the study of TAK1 in the field of RCD, and among them, 25 have published more than or equal to 5 papers. According to **Table 3**, Jun Tsuji has the most published papers (n=26, 3.25%), followed by Kunihiro Matsumoto (n=16, 2.00%) and Shizuo Akira (n=14, 1.75%). **Figure 4A** shows the co-authorship map of these authors. The size of the node denotes the number of publications, and the connecting line between the nodes shows the two authors’ collaboration. The primary publication date of the author’s document is indicated by the node’s color, with yellow suggesting that the author has more recent alliances in the field. As can be seen from the figure, Shizuo Akira and Hiroaki Sakurai, as well as Xu, Xiulong, and Sun, Jing, as the top ten authors in terms of the number of published papers, have worked closely together.

**Table 3:** TOP 10 authors and co-cited authors of TAK1 in the field of RCD.

Rank	Author	Documents	Citations	Co-cited author	Citations
1	Jun Tsuji	26	1545	Jun Tsuji	182
2	Kunihiro Matsumoto	16	1831	Hiroaki Sakurai	175
3	Shizuo Akira	14	1095	Sajedah M Hindi	152
4	Bharat B Aggarwal	11	1492	Kouichi Yamaguchi	143
5	Hiroaki Sakurai	9	254	Emily Omori	135
6	Xu, Xiulong	8	116	Giichi Takaesu	132
7	Sun, Jing	8	109	Jae-Hyuck Shim	111
8	Gautam Sethi	7	1114	Wang, Cuicui	108
9	Emily Omori	7	384	Michael Karin	92
10	Li, Lei	7	155	Yves Dondelinger	79

We have built a collaborative network of authors based on the number of documents cited (**Figure 4B**). The size of the node represents the number of times it has been referenced. When two different authors are cited for their work at the same time, this is known as co-cited authorship analysis. The more times authors are cited together, the more strongly related they are in the eyes of citers. Among the 18910 co-cited authors, 22 have more than 50 co-citations, with Jun Tsuji being the most frequently mentioned. And, Jun Tsuji and Emily Omori are both top 10 authors and top 10 co-cited authors (**Table 3**).

### 3.4 Distribution of journals

The analysis of the distribution of publication sources helps to pinpoint high-impact journals in this field. Based on Vosviewer’s analysis in **Table 4**, all of these 801 papers that addressed TAK1 in the field of RCD are published in 312 journals. *Journal of Biological Chemistry* had the highest output (n=38, 4.74%), followed by *Cell Death & Disease* (n=18, 2.25%). **Table 4** shows that the most cited journal in total is *Journal of Biological Chemistry* (3438), followed by *EMBO Journal* (1470) and *Cell Death and Differentiation* (1303). Among the top 10 journals, *EMBO journal* had the highest impact factor. The top 10 journals published 158 papers, accounting for 19.73% of all publications.

**Table 4:** Top 10 cited journals of TAK1 in the field of RCD.

Rank	Journal Tittle	Count(%)	Total citations	Quartile in category	IF (JCR 2022)
1	Journal of Biological Chemistry	38 (4.74)	3438	Q2	4.8
2	Cell Death & Disease	18 (2.25)	495	Q1	9.0
3	Plos One	18 (2.25)	459	Q2	3.7
4	Cell Death and Differentiation	15 (1.87)	1303	Q1	12.4
5	Proceedings of the National Academy of Sciences of the United States of America	14 (1.75)	1203	Q1	11.1
6	Molecular and Cellular Biology	13 (1.62)	1013	Q2	5.3
7	Journal of Immunology	12 (1.50)	447	Q2	4.4
8	Biochemical and Biophysical Research Communications	12 (1.50)	299	Q3	3.1
9	EMBO Journal	9 (1.12)	1470	Q1	11.4
10	International Immunopharmacology	9 (1.12)	105	Q1	5.6

**Figure 5:**
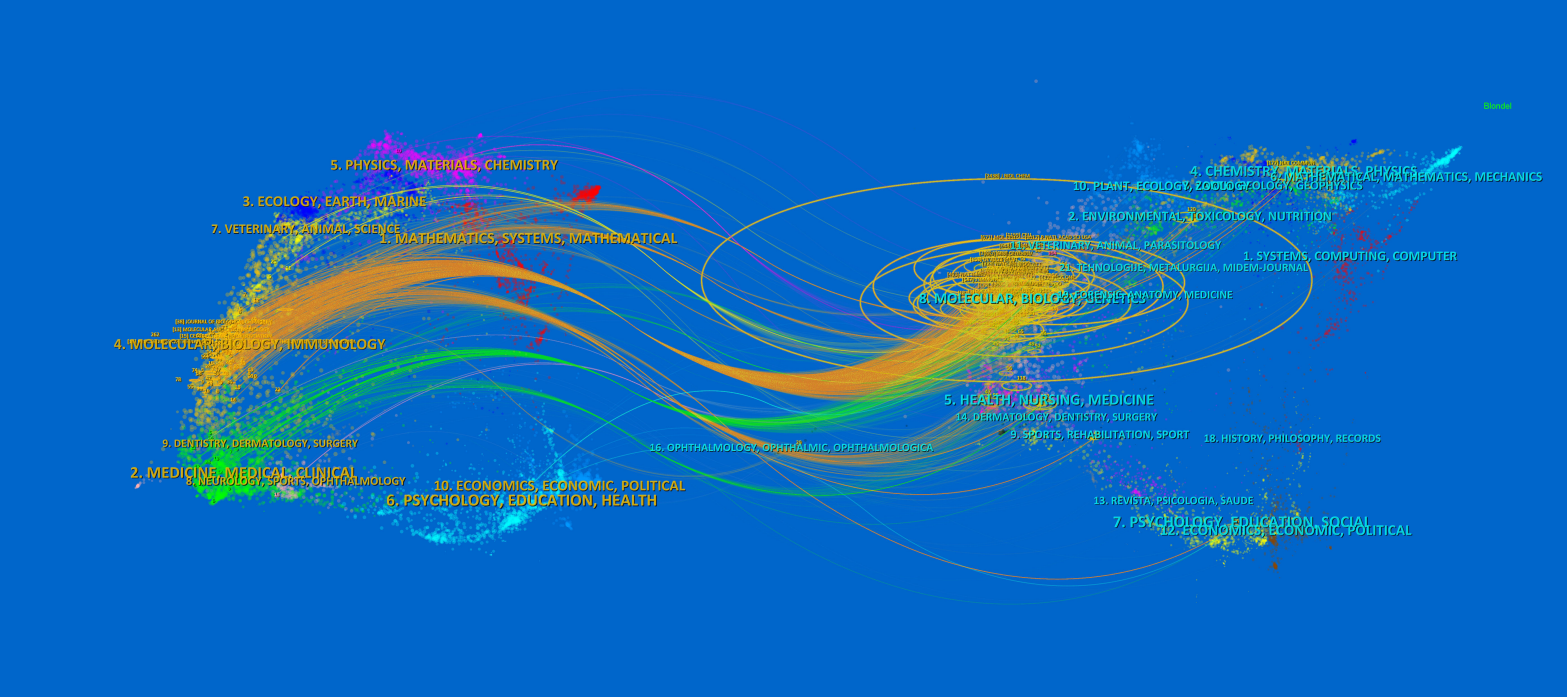
The dual-map overlay of journals of TAK1 in RCD field. The left half is the distribution of the subjects to which the citing journals belong, representing the current state of research. The right half is the clusters of cited journals, representing the research base. The wave curve shows the relationship between the current state of research and the research base, and the size of the circle indicates the number of articles published in each subject.

The dual-map overlay of journals shows the citation relationship between journals with different topics, which visualizes the distribution and citation of important journals in the research field and allows for a quick grasp of the disciplinary interaction characteristics (42). As shown in **Figure 5**, the left half is the distribution of the subjects to which the citing journals belong, representing the current state of research. The right half is the cluster of cited journals, representing the research base. The wave curve shows the relationship between the current state of research and the research base, and the size of the circle indicates the number of articles published in each subject. The primary citation path is from Molecular/Biology/Genetics and Medicine/Medical/Clinical journals to Molecular/Biology/Immunology journals (**Figure 5**).

### 3.5 Co-cited references and reference burst

**Figure 6:**
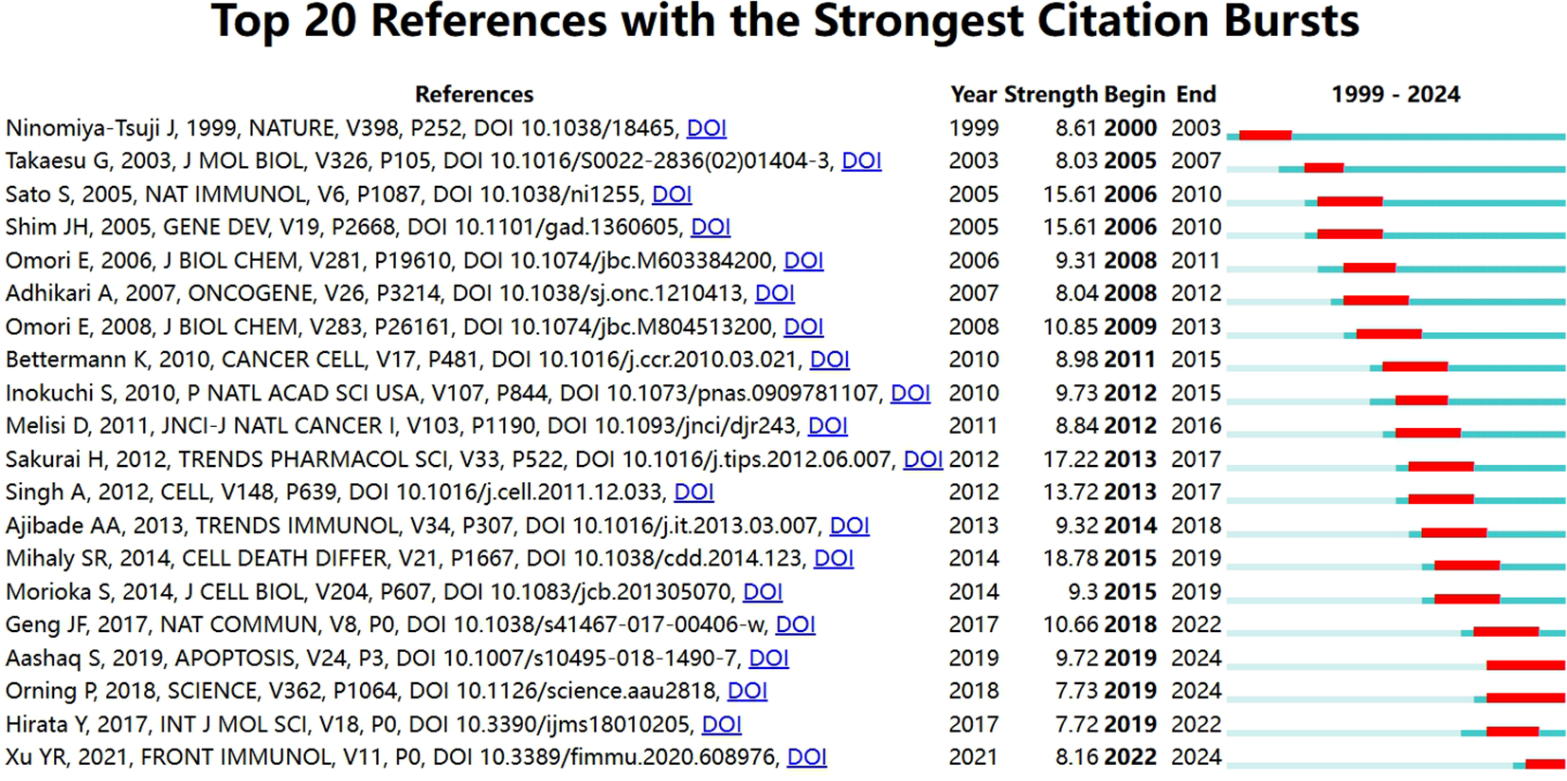
Top 20 references with the strongest citation bursts.

If multiple articles are cited by the same article, then we assume that these co-cited articles are related. The greater frequency of these co-cited references suggests that the intrinsic relationship between these articles may be stronger. In our study, there are 24,811 co-cited references on the research of TAK1 in the RCD field. **Table 5** demonstrates the top 10 co-cited references. Among them, the most co-cited reference (n=129) is an article published in *Nature Immunology* by Shintaro Sato et al. in 2005. The citation burst refers to the high frequency of citations that have been highlighted over a period of time (43). Based on CiteSpace, we found the 20 most references with strong citation bursts (**Figure 6**). On the timeline, the blue portion indicates when the literature was published, and the red portion indicates the period of the citation burst. The reference with the strongest citation burst (strength=18.09) was titled “TAK1 control of cell death”, authored by S R Mihaly et al. with citation bursts from 2014 to 2019.

**Table 5:** Top 10 co-cited references of TAK1 in the field of RCD.

Rank	Reference	Journal	Published year	Citations	Total link strength	Refs
1	Essential function for the kinase TAK1 in innate and adaptive immune responses	Nature Immunology	2005	129	970	(44)
2	Identification of a member of the MAPKKK family as a potential mediator of TGF-beta signal transduction	Science	1995	124	847	(1)
3	The kinase TAK1 can activate the NIK-I kappaB as well as the MAP kinase cascade in the IL-1 signaling pathway	Nature	1999	111	807	(100)
4	TAK1 is a ubiquitin-dependent kinase of MKK and IKK	Nature	2001	901	574	(45)
5	TAK1, but not TAB1 or TAB2, plays an essential role in multiple signaling pathways *in vivo*	Genes & Development	2005	98	816	(49)
6	TAK1 control of cell death	Cell Death and Differentiation	2014	81	442	(46)
7	A resorcylic acid lactone, 5Z-7-oxozeaenol, prevents inflammation by inhibiting the catalytic activity of TAK1 MAPK kinase kinase	The Journal of Biological Chemistry	2003	74	442	(101)
8	Targeting of TAK1 in inflammatory disorders and cancer	Trends in Pharmacological Sciences	2012	74	507	(4)
9	TAB1: an activator of the TAK1 MAPKKK in TGF-beta signal transduction	Science	1996	62	483	(102)
10	Cell type-specific function of TAK1 in innate immune signaling	Trends in immunology	2013	56	612	(5)

### 3.6 Keyword analysis of research hotpots

**Figure 7:**
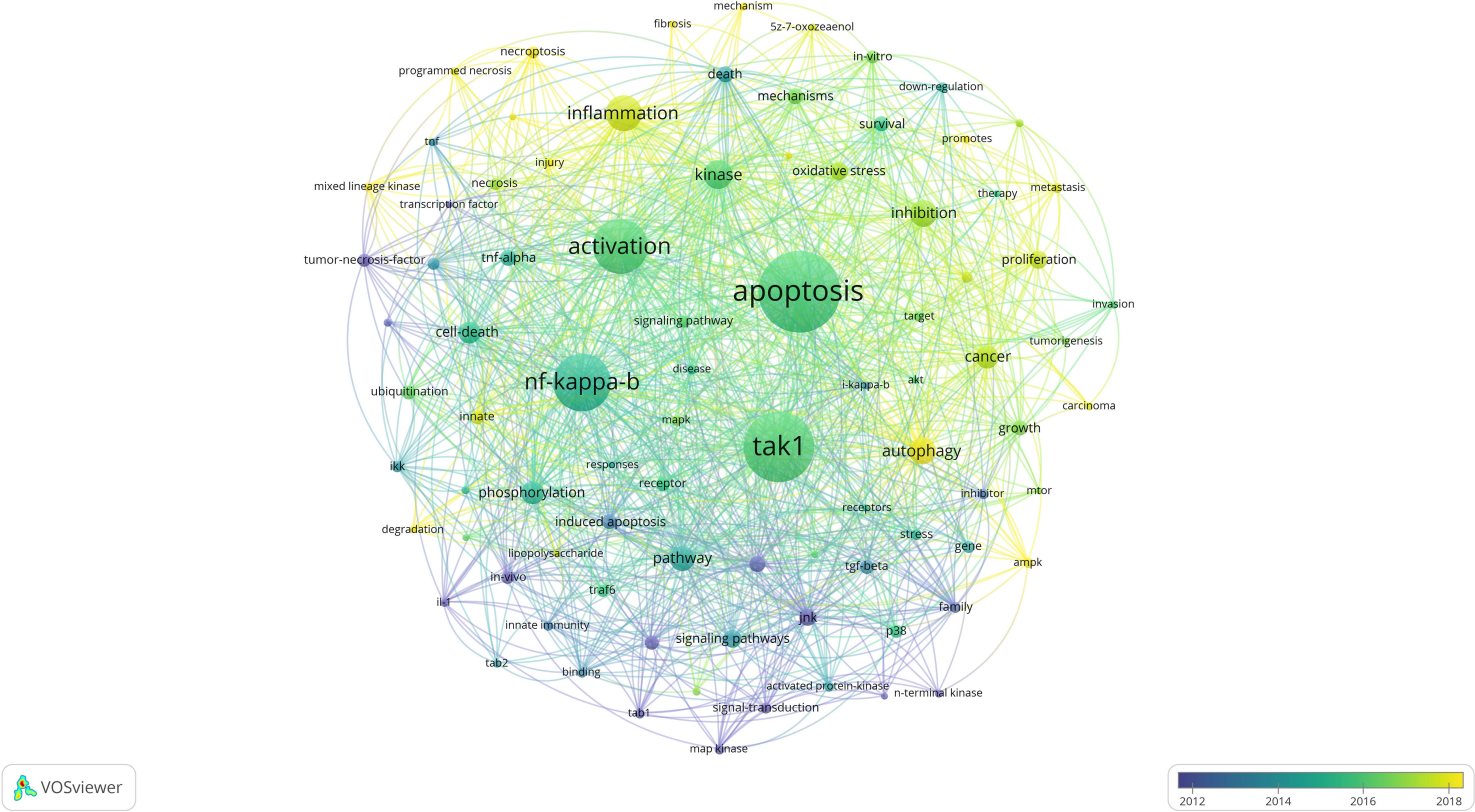
The network visualization map of keywords. The thickness of the connecting lines between the nodes reflects the strength of the interaction. The color of the node represents the average year of publication for the country which the node represents.

**Figure 8:**
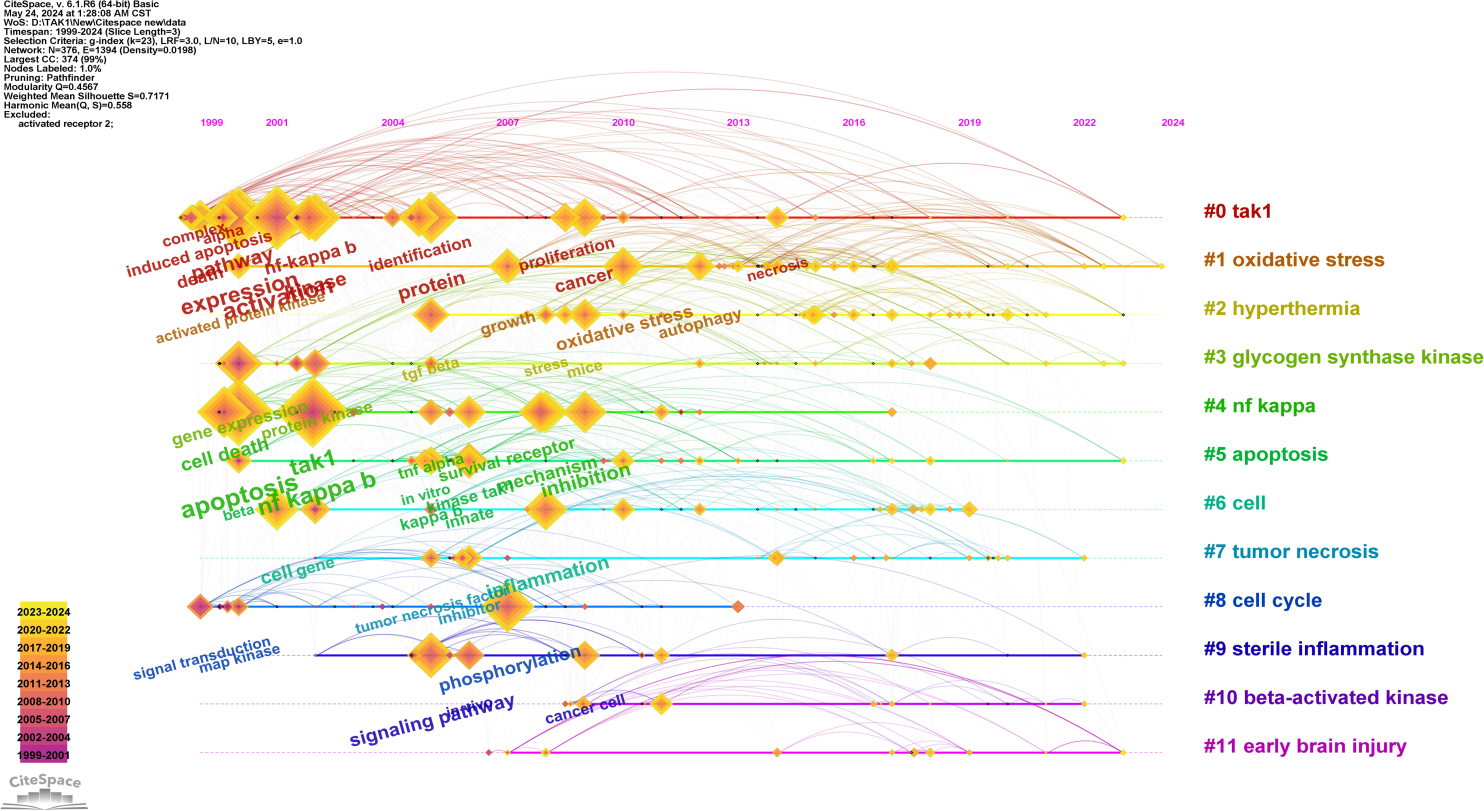
Timeline view of keywords analysis. On the timeline, the blue portion indicates when the literature was published, and the red portion indicates the period of the citation burst.

**Figure 9:**
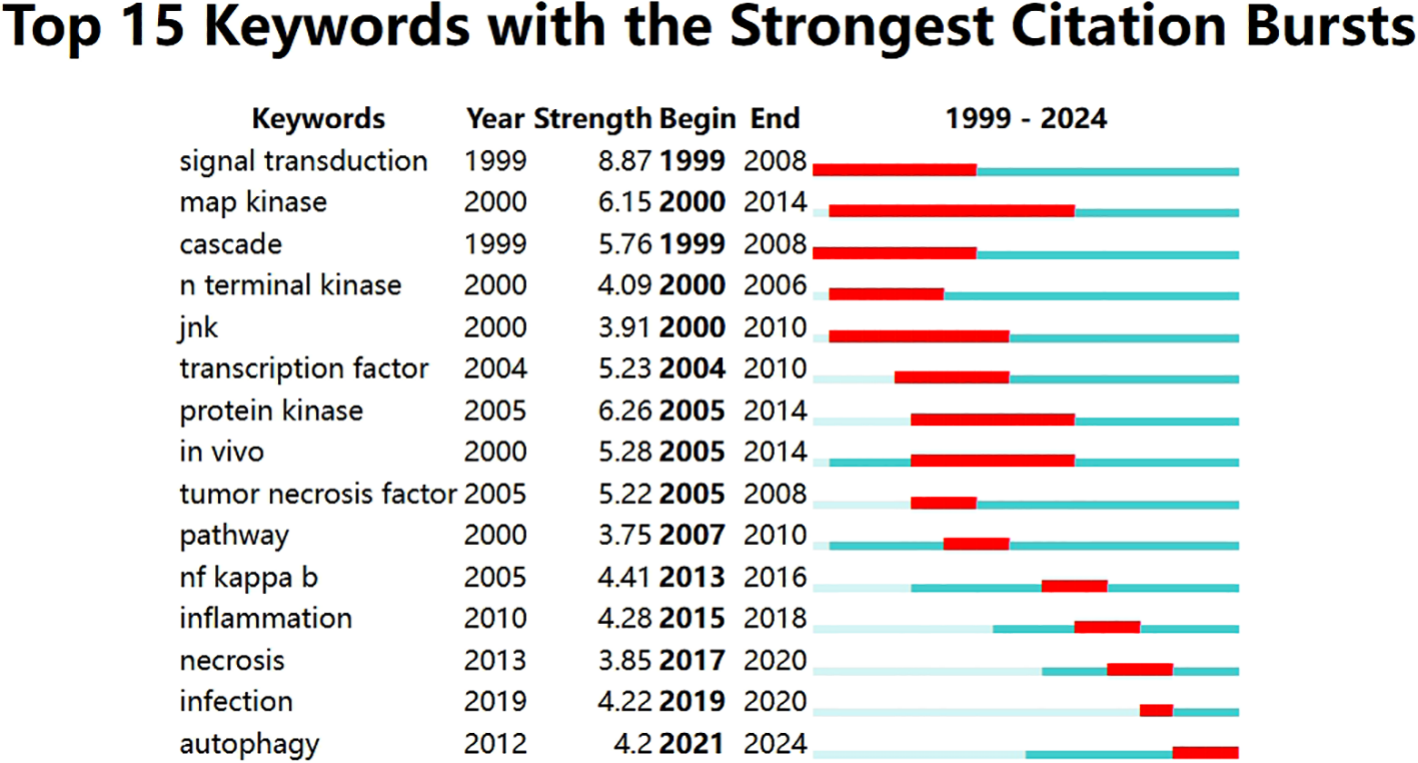
Top 15 keywords with the strongest citation bursts.

Keywords are tangible manifestations of the content of a paper. We may summarize research themes in specific fields and investigate hotspots and directions using keyword analysis. We can swiftly identify research hotspots in a specific topic by using term co-occurrence analysis. **Table 6** shows the top 15 high-frequency keywords in research of TAK1 in RCD and the keywords with the strongest citation burst. Among these keywords, apoptosis and NF-κB appear more than 200 times, which represent the main research topics. The keywords with the strongest citation burst are also displayed as an overlay map in **Figure 7**, with the color representing the average year of publication. As we can see, autophagy, 5Z-7-oxozeaenol, inflammation, and oxidative stress are emerging fields. The timeline viewer is based on the interaction of keywords in a particular field, which aids in determining the evolutionary trajectory and stage characteristics of the study field. **Figure 8** shows the timeline viewer drawn in this study based on CiteSpace, which visualizes the stage-specific hotspots and development direction of TAK1 in RCD field research from a time-dimensional perspective. **Figure 9** shows the top 15 keywords with the strongest citation bursts. We can see that signal transduction and tumor necrosis factor have strong citation bursts. In particular, the keyword oxidative stress remains in burst until 2024. Notably, TAK1 has been found to be closely associated with various diseases in RCD studies (**Figures 8**, **9**), such as cancers, inflammatory diseases, and infections.

**Table 6:** Top keywords related to TAK1 in cell death.

Rank	Keyword	Occurrences	Total link strength	Keyword with the strongest citation burst	Burst	Centrality
1	Apoptosis	323	1024	Signal transduction	8.87	0.03
2	TAK1	270	927	Protein kinase	6.26	0.04
3	NF-κB	202	649	MAPK	6.15	0.03
4	Activation	189	665	Cascade	5.76	0.02
5	Inflammation	106	368	*In vivo*	5.28	0.07
6	Autophagy	73	266	Transcription factor	5.23	0.04
7	Inhibition	73	248	Tumor necrosis factor	5.22	0.06
8	Cancer	61	230	NF-κB	4.41	0.19
9	Phosphorylation	58	196	Inflammation	4.28	0.05
10	Cell-death	55	182	Infection	4.22	0.01
11	Proliferation	42	168	Autophagy	4.2	0.05
12	Jnk	39	148	N-terminal kinase	4.09	0.01
13	TNF-alpha	38	135	JNK	3.91	0.06
14	Survival	33	131	Necrosis	3.85	0.01
15	Ubiquitination	32	131	Pathway	3.75	0.06

## 4 Discussion

### 4.1 General bibliometric information

The bibliometric analysis of this study is based on 801 refined documents from 1026 organizations with 4663 authors in the WOSCC database from 1999 to 2024. After 2005, the number of annual publications increased rapidly, which indicates the research of TAK1 in RCD is in an explosive period, and that related research has attracted more and more scholars’ attention. For example, among the Top 10 co-cited references of TAK1 in the field of RCD, there were eight papers in the past two decades, and the studies on the functions of TAK1 in the innate and adaptive immune responses foster the exploration of its roles in different diseases (4, 5, 44). In China, the United States, and Japan, researchers contribute the most papers in this field. In addition, the total citations and total link strength of documents from the United States are the highest. For example, a study from University of Texas Southwestern Medical Center received 1657 citations around the world (45). Among the top 15 research institutions, nine are from China and three are from the USA. However, there is relatively little cooperation between Chinese, American, and Japanese institutions, which is not conducive to the development of the field in the long run.

From the perspective of the author, Jun Tsuji, Kunihiro Matsumoto, and Shizuo Akira are the authors with the highest number of publications. In 2005, the team of Jun Tsuji and Shizuo Akira published a paper on TAK1 in the immune response, and is the most co-cited paper in the field (44). In terms of co-cited authors, Jun Ninomiya Tsuji having the highest number of citations, followed by Hiroaki Sakurai and Sajedah M Hindi. These contributions established the important impacts of Japanese authors in this field.

Concerning the top journals, *Journal of Biological Chemistry* (3438) is the most referenced journal overall (1303). These included scientific journals that mostly covered cell biology, immunology, and medical topics. This is in line with the dual mapping study, which shows that the most common citation paths for TAK1 in RCD research are connected to immunology and molecular biology (46).

The knowledge base might be represented partially by co-cited references referenced by the relevant research team (40, 47). When it comes to the top 10 co-cited references of TAK1 in RCD, two are reviews (4, 46), and eight are research papers in which the main themes are related to cell growth and differentiation, immunity, and inflammatory signaling (44, 48–50). And the research began focused on the associations between TAK1 and necrosis, autophagy and infection after 2005. As for the reference burst analysis, three references are still bursting and deserve our attention: they are mainly related to the involvement of TAK1 in multiple signaling pathways and regulate cell fate toward different endings (51–53).

### 4.2 The research hotspots and trending

Based on keyword co-occurrence analysis, the distribution and development of different research hotspots in a particular field can be understood. In the current studies, the main keywords include “apoptosis”, “NF-κB”, “activation”, “inflammation”, “autophagy”, “inhibition”, “cancer”, “phosphorylation”, and “cell death”. We attempted to objectively analyze the hotspots and frontiers of TAK1 research in this study by analyzing keyword co-occurrence, keyword overlay, chronology, and keyword burst to help characterize TAK1 research hotspots and development horizons in the field of RCD.

#### 4.2.1 TAK1 is involved in the regulation of the TNF signaling pathway

TNFα is a family of versatile cytokines that promote inflammation which are strongly linked to a wide range of signaling pathways that result in various outcomes such as cell survival, apoptosis, and inflammation (54, 55). In response to TNFα stimulation, molecules including TNFα receptor-associated factor 2 (TRAF2) and TRAF5, TNFα receptor type 1-associated death structural domain protein (TRADD), and receptor-interacting protein kinase 1 (RIPK1) form a complex. RIPK1 catalyzes the synthesis of polyubiquitin chains, and the RIPK1 polyubiquitin chain binds to the TAK1-TABs complex via TAB2 and phosphorylates TAK1 (56–58).

Different post-translational modifications (PTMs) of the TAK1-TABs complex play an important role in the signaling regulation. TAK1, which is activated phosphorylates the downstream NF-κB kinase inhibitor β (IKKβ). Once IKKβ gets activated, it phosphorylates the IκB protein, and it is subsequently degraded by the proteasome, leading to activation of NF-κB and transcription of its target gene (59–61). In addition to NF-κB signaling, TAK1 also stimulates the transcription of pro-survival and pro-inflammatory cytokine genes by activating the p38 and JNK signaling pathways (62). After the inhibition of TAK1 phosphorylation, activation of caspase8 and caspase3 was observed upon TNFα stimulation, suggesting that TAK1 inhibits caspase cascade activation and is involved in the regulation of apoptosis (63). However, even with caspase8 inhibition, TAK1-deficient cells still undergo necroptosis toward a lethal outcome upon TNF stimulation (64).

#### 4.2.2 The role of TAK1 in IL-1 signaling pathway

In addition to its role in TNFα signaling, TAK1 also responds to the upstream inflammatory signaling molecule Interleukin-1 (IL-1). IL-1β, a key pro-inflammatory cytokine, is involved in a variety of cellular activities, including cell proliferation, differentiation, and apoptosis (65). Similar to TNFα, IL-1 signaling regulates a variety of inflammatory responses following infection (60). The IL-1 receptor (IL-1R) binds to the corresponding ligands and recruits myeloid differentiation primary response 88 (MyD88), IL-1 receptor-associated kinase 1 (IRAK1) and IRAK4 to form a complex. IRAK1 then binds to TRAF6, an E3 ubiquitin ligase, which promotes K63-linked polyubiquitination and recruits TAB2 to form the TRAF6-TAB2-TAK1 complex to activate TAK1 by phosphorylation at the Thr178 and Thr184 sites (7, 48, 66). The study shows that antagonism of the IL-1 receptor significantly reduces levels of TNF-α, pro-IL-1β and pro-caspase-1 and inhibits pyroptosis (67).

#### 4.2.3 The role of TAK1 in TGFβ signaling pathway

TGFβ is a pleiotropic cytokine that controls multiple cellular activities via both classical (Smad-dependent) and non-classical (Smad-independent) mechanisms (68). Non-canonical TGFβ pathways include the MAPK and NF-kB pathways. TRAF6 can be delivered to the TGFβ receptor. As previously described, upon binding to the TGFβ receptor, TRAF6 is auto-ubiquitinated, which in turn leads to K63-linked polyubiquitination of TAK1 at Lys34 and activation of TAK1 (69, 70). The activation of downstream pathways is then mediated by activated TAK1. Inhibition of TAK1 expression can block the TGFβ/p38 MAPK signaling pathway and p38 phosphorylation, and decrease Bax and caspase3 expression thereby reducing apoptosis (71). In addition, TAK1 mediates excessive autophagy through the MAPK signaling pathway in chemotherapeutic drug-induced acute kidney injury (72).

#### 4.2.4 TAK1 in the regulation of PANoptosis

PANoptosis is an inflammatory RCD that combines the key features of three major RCD pathways (pyroptosis, apoptosis, and necroptosis), as represented by the acronym PAN (73). When PANoptosis is triggered within the tissue, it shows complex morphological changes related to RCD that can be detectable through positive staining for PI, EthD-III, and TUNEL staining, distinct from those seen in pyroptosis, apoptosis, or necroptosis individually. The regulation of PANoptosis is complex and has not been fully elucidated. This complicated modulation is achieved through the PANoptosome, a multi-protein complex that contains key proteins responsible for the activation of cellular pyroptosis, apoptosis, and necroptosis (74). TAK1 deficiency causes PANoptosis in macrophages, and activation of PANoptosome components is also seen (75, 76). The excessive activation of TNFα and the NF-κB was also detected in macrophages undergoing PANoptosis, indicating that TAK1 is involved in the occurrence of PANoptosis via TNFα and NF-κB signaling pathway. In addition, PANoptosis has been shown to play a role in cancers, including thyroid cancer, hepatocellular carcinoma, and colon adenocarcinoma (77–79). Evidence from bioinformatic analysis and experimental validation support the critical role of PANoptosis in cancer, highlighting its potential as a target for therapeutic intervention (80). Also, molecular clustering studies based on PANoptosis showed that it can be used to predict patient survival and indicate the intricacies of the tumor microenvironment (78). In colorectal cancer, deficiency of interferon regulatory factor 1 (IRF1) in mice could reduce the activation of the PANoptosome and PANoptosis, showing more hypersusceptible to colorectal tumorigenesis (81). A further study indicated that IRF1-deficient cells showed reduced cell death compared with wild-type cells in response to treatment with a TAK1 inhibitor plus lipopolysaccharide (82). It suggests a regulatory role of the IRF1-TAK1 axis in PANoptosis and the potential value of targeting this axis for treating tumorigenesis. The studies on the regulatory roles of TAK1 in PANoptosis provide the potential to design a powerful strategy that simultaneously targets multiple cell death pathways, potentially overcoming resistance mechanisms and improving treatment efficacy (75).

#### 4.2.5 The role of TAK1 in cancers and inflammatory disorders

Numerous investigations have revealed that TAK1 has a significant role in the incidence and progression of cancers (74, 83, 84), infections (85, 86), metabolic disorders (87, 88), and neurological system diseases (89).

Activation of TAK1/NF-κB signaling by CXCR2 receptors enhances oncogenic and metastatic potential both *in vitro* and *in vivo* (84). In colon cancer, Anurag et al. demonstrated that TAK1 promoted the survival of KRAS-dependent colon cancer cells (90). In addition, the promising therapeutic value of targeting TAK1 was validated by 5Z-7-oxozeaenol, a potent and selective TAK1 kinase inhibitor. Notably, inhibition of TAK1 was found to induce apoptosis in KRAS-dependent colon cancers, further emphasizing its significance as a therapeutic target in this context. The genetic silencing or inhibition of TAK1 also has been shown to promote the progression of prostate cancer (91). The loss of TAK1 has been found to play an active role in cancer metastasis. TAK1 deficiency enhances metastasis and causes epithelial-mesenchymal transition in cancer cells in a ROS-dependent way in skin squamous cell carcinoma (92).

TAK1 regulates autophagy in chemotherapeutic drug-induced acute kidney damage via the MAPK signaling pathway (72). In the animal model of collagen-induced arthritis (CIA), daily doses of takinib, a TAK1 inhibitor, significantly reduced clinical joint inflammation in mice (93). 5Z-7-oxozeaenol slows the progression of Nonalcoholic steatohepatitis by inhibiting the activation of the mitogen-activated protein kinase (MAPK) signaling pathway via suppressing TAK1 (94). In addition to inflammation, TAK1 is involved in other diseases. Inhibition of TAK1 by 5Z-7-oxozeaenol effectively reduces gout symptoms, which may be related to the IL-1 signaling pathway (95). And inhibition of TAK1 by 5Z-7-oxozeaenol blocked TNFα-mediated NF-κB signaling, which could reduce retinal neovascularization (96).

The activation of TAK1 plays an important role in innate immunity and inflammatory responses. In inflammatory lung injury, the mutant form of TAK1, wherein Ser412 is replaced with alanine (TAK1 S412A), demonstrates an inhibition in downstream signaling (97). Additionally, activated focal adhesion kinase (FAK) has been observed to directly engage with TAK1, facilitating the phosphorylation of the Ser412 site and subsequently modulating inflammatory responses associated with lung injury. Moreover, mice with hepatocyte-specific deletion of TAK1 exhibit liver inflammation, fibrosis, and a heightened susceptibility to liver cancer progression (98). Notably, TAK1-deficient hepatic cells undergo spontaneous apoptosis, a process that can be further exacerbated by TNFα stimulation.

Metformin activates chaperone-mediated autophagy and degrades Aβ through activation of TAK1-IKKα/β signaling to ameliorate pathological symptoms in Alzheimer’s mice (99). *In vitro* and *in vivo* experiments show that inhibiting TAK1 blocks the MAPK signaling pathway and NF-κB signaling pathway, reducing neuroinflammation and apoptosis to exert a neuroprotective effect (89, 99). The development of TAK1-specific inhibitors that can cross the blood-brain barrier, provides new opportunities for the treatment of central nervous system diseases.

### 4.3 Strengths and limitations

This study is the first bibliometric research to provide a retrospective and inclusive visual depiction of the research trends and advancements concerning TAK1 in RCD, analyzing publications spanning from 2009 to 2024. However, certain limitations stem from the data acquisition. Firstly, our search was confined to the WoS Core Collection database, potentially excluding relevant studies not indexed within this repository and causing an underestimation of the sample size. Also, the language of articles was constrained to English, which might result in the proportion of contribution from the non-English areas being lower than expected. At last, the dynamic nature of the database means that our study might have overlooked some of the latest research findings due to ongoing updates.

## 5 Conclusion

The study of TAK1 involved in RCD regulation has important research value and broad application prospects in human diseases. Based on bibliometric and visualized analysis by CiteSpace and VOSviewer, we found that research in this field has risen rapidly in the past two decades. From a global point of view, China, the United States, and Japan are leading the research. Among the research institutions, Osaka University is the one with the highest impact. There is also a need to strengthen cooperation and exchange between different countries and institutions. This bibliometric and visualized study depicts the research status of TAK1 in RCD and proposes ideas for guiding their future investigations in molecular mechanisms and therapeutic strategies in this field.

## Data Availability

The original contributions presented in the study are included in the article/supplementary material. Further inquiries can be directed to the corresponding authors.
